# A Factor Analysis of Global GABAergic Gene Expression in Human Brain Identifies Specificity in Response to Chronic Alcohol and Cocaine Exposure

**DOI:** 10.1371/journal.pone.0064014

**Published:** 2013-05-22

**Authors:** Mary-Anne Enoch, Basel Baghal, Qiaoping Yuan, David Goldman

**Affiliations:** Laboratory of Neurogenetics, National Institute on Alcohol Abuse and Alcoholism, National Institutes of Health, Bethesda, Maryland, United States of America; Yale University, United States of America

## Abstract

Although expression patterns of GABAergic genes in rodent brain have largely been elucidated, no comprehensive studies have been performed in human brain. The purpose of this study was to identify global patterns of GABAergic gene expression in healthy adults, including *trans* and *cis* effects in the GABA_A_ gene clusters, before determining the effects of chronic alcohol and cocaine exposure on gene expression in the hippocampus. RNA-Seq data from ‘BrainSpan’ was obtained across 16 brain regions from postmortem samples from nine adults. A factor analysis was performed on global expression of 21 GABAergic pathway genes. Factor specificity for response to chronic alcohol/cocaine exposure was subsequently determined from the analysis of RNA-Seq data from postmortem hippocampus of eight alcoholics, eight cocaine addicts and eight controls. Six gene expression factors were identified. Most genes loaded (≥0.5) onto one factor; six genes loaded onto two. The largest factor (0.30 variance) included the chromosome 5 gene cluster that encodes the most common GABA_A_ receptor, α_1_β_2_γ_2_, and genes encoding the α_3_β_3_γ_2_ receptor. Genes within this factor were largely unresponsive to chronic alcohol/cocaine exposure. In contrast, the chromosome 4 gene cluster factor (0.14 variance) encoding the α_2_β_1_γ_1_ receptor was influenced by chronic alcohol/cocaine exposure. Two other factors (0.17 and 0.06 variance) showed expression changes in alcoholics/cocaine addicts; these factors included genes involved in GABA synthesis and synaptic transport. Finally there were two factors that included genes with exceptionally low (0.10 variance) and high (0.09 variance) expression in the cerebellum; the former factor was unaffected by alcohol/cocaine exposure. This study has shown that there appears to be specificity of GABAergic gene groups, defined by covariation in expression, for response to chronic alcohol/cocaine exposure. These findings might have implications for combating stress-related craving and relapse.

## Introduction

Gamma-aminobutyric acid (GABA) is the principal inhibitory neurotransmitter in the vertebrate central nervous system (CNS). It is estimated that approximately 40% of mammalian CNS synapses have GABA receptors of which there are two classes: GABA_A_ receptors that are ligand-gated, chloride ion channels that confer fast synaptic inhibition, and metabotropic GABA_B_ receptors that are coupled to downstream calcium and potassium channels by G proteins. GABA is involved in CNS development and organization, learning and memory, anxiety and addiction [1]. GABA_A_ receptors undergo allosteric modulation by several drugs including ethanol, benzodiazepines (BZs), barbiturates, anesthetics and endogenous neurosteroids [1].

GABA_A_ receptors are composed of five subunits, each of which has several isoforms (α_1–6_, β_1–3_, γ_1–3_, δ, ε, θ, π, ρ_1–3_) [2]. Most receptors consist of two α, two β and one γ subunits [3]. Preclinical studies have identified three main subunit combinations: α_1_β_2_γ_2_ (60%), α_2_β_3_γ_2_ (15%), and α_3_β_3_γ_2_ (10%) [4]–[5]. Studies in rodents and non-human primates have shown that the expression of the various subunit isoforms varies across brain locations and during development. The receptor subunit composition determines distinct pharmacological and electrophysiological properties [6]. It has been shown that in non-human primates, the anxiolytic and muscle relaxant effects of BZ-like drugs appear to involve receptors including α_2,_ α_3_ and α_5_ subunits whereas the ataxic and sedative properties of BZ-like drugs are mediated by α_1_ subunits [7]–[8]. Receptors including α_4_ and α_6_ subunits are BZ insensitive.

Genes for 14 of the 19 GABA_A_ receptor subunit isoforms are clustered in four chromosomal regions: 4p13–q11 (α_2,_ α_4,_ β_1,_ γ_1_), 5q34–q35 (α_1,_ α_6,_ β_2,_ γ_2_), 15q11–q13 (α_5,_ β_3,_ γ_3_), and Xq28 (α_3,_ θ,ε) [2]. Alternative splicing has been demonstrated for most of the GABA_A_ receptor subunit isoforms indicating complexity in gene expression [9]–[10]. There is considerable conservation and linkage disequilibrium extending across long stretches of sequence in the chromosome (chr) 4 and 5 clusters [1]. This extensive and conserved pattern of gene clustering was established early in vertebrate evolution, after divergence from the invertebrates [11]–[12]. It is thought that the four gene clusters originated from duplications of (and within) an ancestral gene cluster containing single genes encoding one α, one β and one γ subunit [13].

A key question is whether the expression of genes within each chromosomal cluster are correlated (*cis* effects) and whether there is correlation of gene expression between GABAergic genes that are located in different regions (*trans* effects). In order to address this issue we obtained RNA-Seq data from postmortem samples of 16 brain regions from nine healthy men and women aged 18–40 who died suddenly. The RNA-Seq data was obtained from BrainSpan, a publicly available resource (http://www.brainspan.org/). In addition to the GABA_A_ subunit genes found in several gene clusters, we included genes encoding the two GABAB receptor subunits (*GABBR1, GABBR2*), genes encoding proteins involved in GABA synthesis (*GAD1*, *GAD2*) and genes encoding proteins involved in GABA regulation (*GPHN*, *SLC6A1* and *PRAF2*). A factor analysis was performed to identify patterns of *cis* and trans correlations in gene expression.

The second aim of the study was to determine whether some of the factors (groups of genes defined by covariation in expression in healthy individuals) identified from the BrainSpan RNA-Seq data, might show changes in expression in response to chronic exposure to alcohol and cocaine whereas other factors might be unaffected by exposure to alcohol or drug. In order to do this we looked at gene expression data from an earlier RNA-Seq study in which we had identified changes in GABAergic gene expression in human postmortem samples of hippocampus from alcoholics, cocaine addicts and controls [14]–[15].

## Materials and Methods

### Databases

There were two sources of RNA-Seq data used in this study; a public resource for factor analyses based on cross-regional influences on GABAergic gene expression and a publicly accessible resource from our lab on effects of alcohol and cocaine exposure on hippocampal gene expression.

#### BrainSpan brain samples

The publicly available data used in this study was derived from BrainSpan, an atlas of the developing human brain, accessed via the Allen Institute for Brain Science’s hosting website http://www.brainspan.org/. Using RNA sequencing, BrainSpan provides unbiased coverage of the complete transcriptome in 16 targeted cortical and subcortical structures (Table 1) in 42 brain specimens to date, spanning pre-natal development to adulthood in both males and females. Details pertaining to sample and tissue acquisition and qualification, processing and dissection, RNA analysis, sequencing and alignment, quality control as well as expression quantification can be found in the BrainSpan Technical White Paper 2011 [16].

**Table 1:** Distinct cortical and subcortical substructures of the adult brain available in BrainSpan for gene expression studies.

	Brain Regions	Abbreviation
1	Hippocampus	HIP
2	Striatum	STR
3	Anterior (rostral) cingulate (medial prefrontal) cortex	MFC
4	Dorsolateral prefrontal cortex	DFC
5	Ventrolateral prefrontal cortex	VFC
6	Orbital frontal cortex	OFC
7	Amygdaloid complex	AMY
8	Cerebellar cortex	CBC
9	Inferolateral temporal cortex (area TEv, area 20)	ITC
10	Posterior (caudal) superior temporal cortex (area TAc)	STC
11	Mediodorsal nucleus of thalamus	MD
12	Posteroinferior (ventral) parietal cortex	IPC
13	Primary somatosensory cortex (area S1, areas 3,1,2)	S1C
14	Primary motor cortex (area M1, area 4)	M1C
15	Primary visual cortex (striate cortex, area V1/17)	V1C
16	Primary auditory cortex (core)	A1C

#### BrainSpan ethics statement

All work was performed according to guidelines for the research use of human brain tissue and with approval by the Human Investigation Committees and Institutional Ethics Committees of the participating institutes.

Only brains with no evidence of malformations or lesions at the macroscopic or microscopic levels were included. All brain tissue samples in BrainSpan met specific selection criteria to ensure consistency and limit potential variation due to disease conditions. In particular, specimens were excluded if excessive drug or alcohol abuse was reported, if the individual had any known neurological or psychiatric disorders or if any prolonged agonal conditions were reported. Other excluding factors included ingestion of neurotoxic substances at the time of death, suicide, severe head injury, significant hemorrhages, prominent vascular abnormalities, tumors, prominent brain lesions, stroke, congenital neural abnormalities, and signs of neurodegeneration. For further details see BrainSpan Technical White Paper 2011 [16].

Table 2 lists details of the nine adult (aged 18–40 years) postmortem brain specimens (samples A – J) that are the focus of this study. Information about ethnicity was not available.

**Table 2:** The nine adult postmortem brain specimens utilized in this study.

Study Sample ID	BrainSpanSample ID	Age(yrs)	Sex	# Brain StructuresCollected
A	H376.X.53	18	M	13^a^
B	H376.X.52	19	F	16
C	H376.XI.60	21	F	16
D	H376.XI.50	23	M	16
E	H376.XI.52	30	F	16
F	H376.XI.53	36	M	16
G	H376.XI.54	37	M	16
H	H376.XI.55	40	M	16
J	H376.XI.56	40	F	15^b^

a no amygdaloid complex, mediodorsal nucleus of thalamus and striatum.

b no anterior (rostral) cingulate (medial prefrontal) cortex.

#### Hippocampal samples from alcoholics, cocaine addicts and controls

Postmortem brain tissue was provided by the University of Miami Brain Bank. Since we used publicly available pathological specimens, our study was exempt from NIH Institutional Review Board (IRB) review. RNA-Seq data from postmortem hippocampus of eight alcoholics, eight cocaine addicts and eight controls were previously generated by our lab [14]–[15] demonstrating neuroadaptive changes. This RNA-Seq data has been deposited in the GenBank database (accession nos. SRA029279 and SRA029275). The GABAergic gene expression data [15] was used to determine whether there might be specificity in the association of any of the six factors, derived from the BrainSpan dataset, with changes in expression in response to chronic alcohol and cocaine exposure.

### GABAergic Genes

The study focused on investigating the expression profiles of genes involved in the GABAergic pathway, predominantly genes encoding the GABA_A_ receptor subunits (Table 3). With the exception of the δ and ρ_3_ subunits, genes encoding the GABA_A_ receptor subunits are clustered in groups of two to five on chromosomes 4, 5, 6, 15, and X. In addition, we also investigated the expression profiles of genes encoding proteins involved in GABA synthesis (*GAD1, GAD2*), neuronal re-uptake of GABA at the synaptic cleft (*SLC6A1*), *GPHN* that encodes a key protein involved in the regulation of synaptic localization of GABA_A_ receptors [17] and *PRAF2*, encoding the PRAF2 protein that is enriched in synaptic vesicles and is thought to be involved in the regulation of vesicular trafficking and neurotransmitter release [18]–[19].

**Table 3:** GABAergic genes included in this study.

Gene Symbol	Name	Chr	Chr Start	Chr End	Strand
			Position	Position	
	**GABA_A_ receptor subunits**				
GABRD	δ delta	1	1940628	1952052	forward
GABRR3	ρ_3_ rho-3	3	99188217	99236838	reverse
GABRG1	γ_1_ gamma-1	4	45732544	45820839	reverse
GABRA2	α_2_ alpha-2	4	45946339	46086813	reverse
GABRA4	α_4_ alpha-4	4	46615674	46691181	reverse
GABRB1	β_1_ beta-1	4	46728052	47123204	forward
GABRB2	β_2_ beta-2	5	160648014	160907708	reverse
GABRA6	α_6_ alpha-6	5	161045236	161062176	forward
GABRA1	α_1_ alpha-1	5	161206775	161259543	forward
GABRG2	γ_2_ gamma-2	5	161427226	161515123	forward
GABRP	π pi	5	170143301	170173628	forward
GABRR1	ρ_1_ rho-1	6	89943942	89984215	reverse
GABRR2	ρ_2_ rho-2	6	90023958	90081686	reverse
GABRB3	β_3_ beta-3	15	24339787	24570028	reverse
GABRA5	α_5_ alpha-5	15	24662959	24777103	forward
GABRG3	γ_3_ gamma-3	15	24799175	25451968	forward
GABRE	ε epsilon	X	150872252	150893807	reverse
GABRA3	α_3_ alpha-3	X	151086290	151370487	reverse
GABRQ	θ theta	X	151557293	151572481	forward
	**GABA_B_ receptor subunits**				
GABBR1	GABABR1	6	29677984	29708941	reverse
GABBR2	GABABR2	9	100090185	100511300	reverse
	**GABA synthesis**				
GAD1	Glutamate decarboxylase 1	2	171381446	171425905	forward
GAD2	Glutamate decarboxylase 2	10	26545242	26633497	forward
	**GABA regulation**				
SLC6A1	GABA transporter 1 (GAT1)	19	54484704	54520286	reverse
GPHN	Gephyrin	14	66043878	66718278	forward
PRAF2	PRA1 domain family, member2	X	48815762	48818606	reverse

### Analyses

This study utilized the RNA-Seq data obtained via the BrainSpan “RNA-Seq summarized to genes” downloadable archive file which contains normalized expression values and meta-data. The archive consists of RPKM (Reads Per Kilobase of exon model per Million mapped reads) values for each gene measured in each of the collected brain structures from each sample. After the archive was downloaded and uncompressed, the relevant information (genes and samples of interest) was extracted and prepared/organized using simple Perl commands. Next, the data was imported into the R package for statistical computing which was used for all subsequent analysis. Box plots were used to visualize expression profiles both sample by sample and gene by gene. Scatter plots and linear regressions were used to visualize correlations in expression which was quantified using the correlation coefficient R^2^. With the exception of the box plots which consistently show Log_2_-transformed RPKM values, no data manipulation was performed.

#### Validation analysis in hippocampal samples from two sources

We have previously used RNA-Seq to quantify mRNA transcripts in postmortem total hippocampus from eight male controls, mean age = 37.5 (SD = 6.1) years obtained from the University of Miami Brain Bank. Full details are available elsewhere [14]–[15]. We compared the expression of the GABAergic genes in our eight hippocampal samples with gene expression in the nine BrainSpan hippocampal samples.

#### Factor analysis

An exploratory factor analysis was performed using the original BrainSpan gene expression values for the 21 GABAergic genes that were expressed in the 16 brain regions. The factor analysis was executed with R version 2.12.1 using the psych (Procedures for Psychological, Psychometric, and Personality Research) package version 1.2.4. We made the decision to include factors with ≥0.05 of the variance. Therefore six factors were extracted that accounted for 0.86 of the variance in total GABAergic gene expression. The fitting method was principal axis factoring (PAF) and the rotation method was set to varimax (orthogonal rotation) since we did not expect the factors to be correlated.

## Results

Our analyses were performed on high quality RNA-Seq data acquired from BrainSpan via the Allen Institute for Brain Science’s hosting website http://www.brainspan.org/together with high quality RNA-Seq data from our earlier study on postmortem hippocampal samples from alcoholics, cocaine addicts and controls [14]–[15]. Figure S1 compares mean (S.E.) expression of the 21 GABAergic genes in hippocampal samples from the controls in our earlier study [15] with the BrainSpan hippocampal samples. It should be noted that the read counts were independently normalized across the two datasets and therefore gene expression levels can only be compared within each dataset and not between the two datasets. Nevertheless Figure S1 shows that the pattern of relative gene expression was virtually the same in the two datasets, thus validating the methodology.

*GABRR1, GABRR2, GABRR3, GABRP* and *GABRE* respectively encoding the GABA_A_ receptor subunits ρ_1_, ρ_2_, ρ_3_, π and ε were barely expressed, if at all, across all 16 brain regions and were therefore excluded from further study.

**Figure 1:**
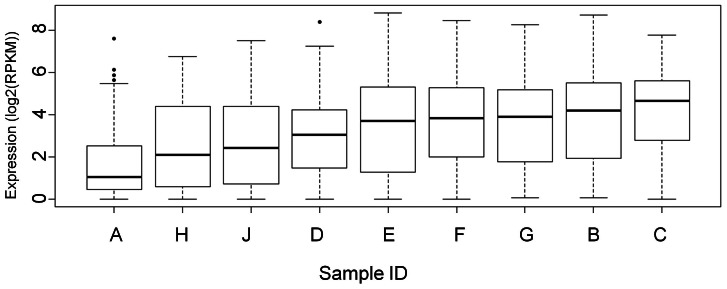
Global expression values across all 21 GABAergic genes in each of the nine BrainSpan individuals. The Y axis indicates log2 RPKM (Reads Per Kilobase of exon model per Million mapped reads) expression values for all 21 genes across all 16 brain regions for each BrainSpan sample. The age and sex of each individual can be found in Table 2.

As shown in Figure 1, the global RPKM expression levels across all the GABAergic genes varied across individuals: sample A (male, 18yrs) had the lowest and sample C (female, 21 years) had the highest global expression values. Since sample A had data available for only 13/16 brain regions, box plots excluding these 3 regions were generated for all 9 samples and yielded the same order of expression level as seen in Figure 1.

Figure S2 displays the expression levels of the 21 GABAergic genes averaged across the 16 brain regions. *GABRA6* and *GABRQ* had the lowest global expression levels and *GABBR1* and *GABRD* had the highest. Figures S3, S4, S5, S6, S7, S8 show the expression levels for each gene in all 16 brain regions.

### Correlation of Expression of 21 GABAergic Genes Across Whole Brain (BrainSpan Samples)

**Figure 2:**
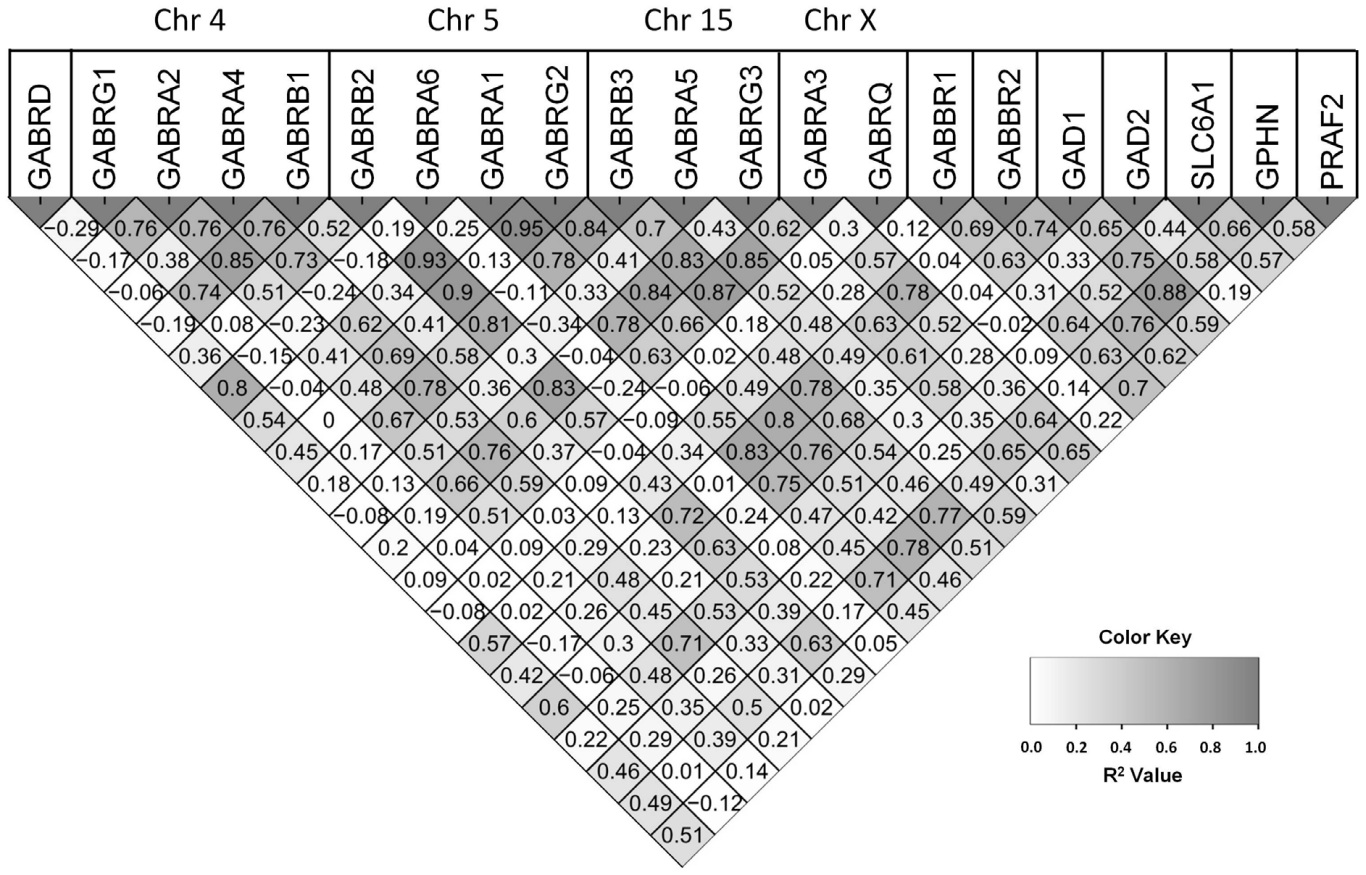
Correlation matrix of gene-gene expression for each GABA receptor subunit and associated GABAergic genes. The heat map is shaded according to the R2 value (see color key). However, the values printed in each cell are the R values so that information about directionality is preserved.

Figure 2 presents the correlation in expression of the 21 GABAergic genes. The scatter plot matrices allow for the identification of genes that show coordinate expression.

### Factor Analysis of 21 GABAergic Genes Across Whole Brain (BrainSpan Samples)

**Figure 3:**
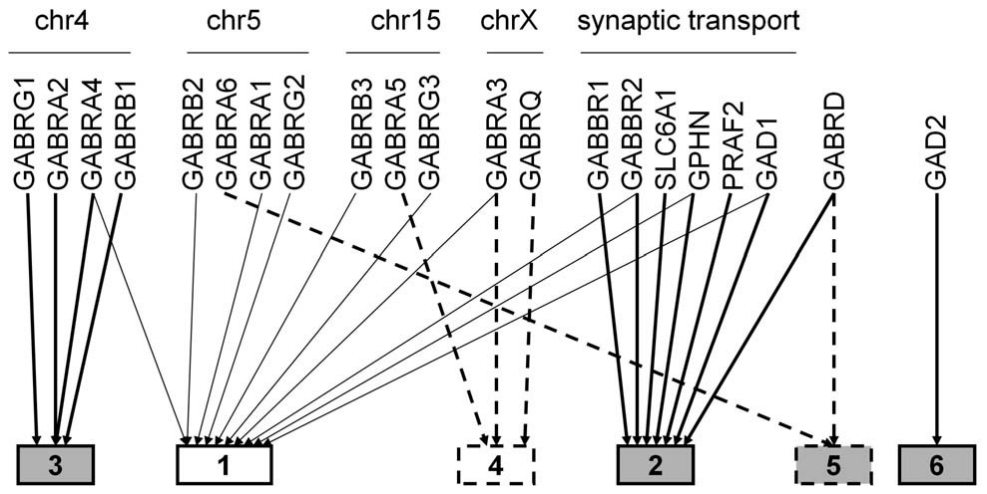
Factor analysis: 6 factor solution. The 6 factors are shown as boxes. Shaded boxes indicate factors influenced by chronic alcohol or cocaine exposure. Dashed outlined boxes indicate the two cerebellar factors. The proportion of the total variance accounted for by each factor is: factor 1∶0.31; factor 2∶0.17; factor 3∶0.14; factor 4∶0.10; factor 5∶0.09; factor 6∶0.06.

The factor analysis identified six factors that together accounted for 0.86 of the variance in total GABAergic gene expression (Table 4, Figure 3). The results presented below are for genes that loaded ≥0.5 on a factor. Out of the total of 21 genes, 15 loaded ≥0.5 onto only one factor. The remaining 6 genes loaded ≥0.5 onto two factors.

**Table 4:** Factor analysis.

Gene	Factor 1	Factor 2	Factor 3	Factor 4	Factor 5	Factor 6
GABRD	0.240	**0.516**	−0.263	−0.091	**0.700**	0.063
GABRG1	−0.101	0.043	**0.969**	−0.020	−0.057	−0.009
GABRA2	0.418	0.075	**0.803**	0.200	−0.136	0.129
GABRA4	**0.680**	0.022	**0.469**	0.178	−0.168	0.347
GABRB1	0.383	−0.005	**0.809**	0.099	−0.095	0.194
GABRB2	**0.904**	0.159	0.197	−0.031	0.173	0.118
GABRA6	0.003	0.139	−0.099	−0.166	**0.979**	0.026
GABRA1	**0.895**	0.338	0.036	−0.015	0.209	0.048
GABRG2	**0.893**	0.298	0.068	0.065	0.112	0.151
GABRB3	**0.789**	0.307	0.252	0.351	−0.099	0.147
GABRA5	0.312	0.210	0.155	**0.951**	−0.192	0.099
GABRG3	**0.809**	0.130	0.283	0.095	−0.023	0.243
GABRA3	**0.628**	0.370	0.098	**0.581**	−0.208	−0.103
GABRQ	−0.054	0.053	0.034	**0.516**	−0.024	0.007
GABBR1	0.292	**0.681**	0.038	0.291	0.281	−0.030
GABBR2	**0.736**	**0.571**	−0.108	0.152	−0.055	−0.059
GAD1	**0.508**	**0.703**	−0.034	0.027	0.132	0.428
GAD2	0.378	0.218	0.298	0.028	0.069	**0.730**
SLC6A1	0.145	**0.813**	0.288	0.012	0.137	0.174
GPHN	**0.555**	**0.598**	0.047	0.193	0.099	0.312
PRAF2	0.252	**0.683**	−0.108	0.423	0.062	−0.054
Variance	30.80%	17.31%	14.10%	9.95%	8.67%	5.57%
Cumulative Variance	30.80%	48.08%	62.18%	72.13%	80.81%	86.38%

Loading ≥0.5 are in bold. The highest factor loading for each gene is indicated with larger font.

#### Factor 1: GABA_A_ subunit genes, including the chr 5 cluster

Ten of the 21 genes loaded onto this factor that accounted for 0.31 of the total variance in GABAergic gene expression. The strongest loading (0.9) was for the chr 5 cluster of genes (*GABRA1, GABRB2, GABRG2*) that together encode the most abundant GABA_A_ receptor subunit group: α_1_β_2_γ_2_. Two chr 15 genes: *GABRB3* and *GABRG3* also had high loadings (0.8) on this factor. The chr 4 gene *GABRA4* and the chr X gene *GABRA3* loaded more modestly (0.7–0.6). In addition, there were loadings for *GABBR2*, the GABAB receptor subunit gene (0.7), *GAD1* (0.5), implicated in GABA synthesis and *GPHN* (0.6), implicated in synaptic localization of GABA_A_ receptors. However it should be noted that *GAD1* and *GPHN* had higher loadings on Factor 2.

#### Factor 2: genes involved in synaptic transport

Seven genes loaded onto this factor that accounted for 0.17 of the total variance in GABAergic gene expression. Six of these genes are implicated in GABA transport across the synaptic cleft. The strongest loading was for *SLC6A1* (0.8) that encodes the neuronal GABA transporter GAT-1. There were also loadings for the two GABAB receptor genes *GABBR1* (0.7) and *GABBR2* (0.6) and for *PRAF2* (0.7), that encodes PRAF2, a protein that is enriched in synaptic vesicles. *GPHN* (0.6) and *GABRD* (0.5) that encodes the extrasynaptic δ GABA_A_ subunit also loaded onto this factor. Finally, *GAD1*, implicated in GABA synthesis, loaded more strongly (0.7) on this factor than on factor 1.

#### Factor 3: chr 4 gene cluster

All four GABA_A_ subunit genes in the chr 4 cluster loaded onto this factor that accounted for 0.14 of the total variance in GABAergic gene expression: *GABRG1* (1.0); *GABRA2* (0.8); *GABRB1* (0.8) and *GABRA4* (0.5). Three of these genes encode the GABA_A_ receptor subunit combination α_2_β_1_γ_1_ that is found almost exclusively in brain regions that form the reward pathway. It is noteworthy that *GABRA4* loaded more strongly on factor 1 (0.7).

#### Factor 4: genes with low cerebellar expression

**Figure 4:**
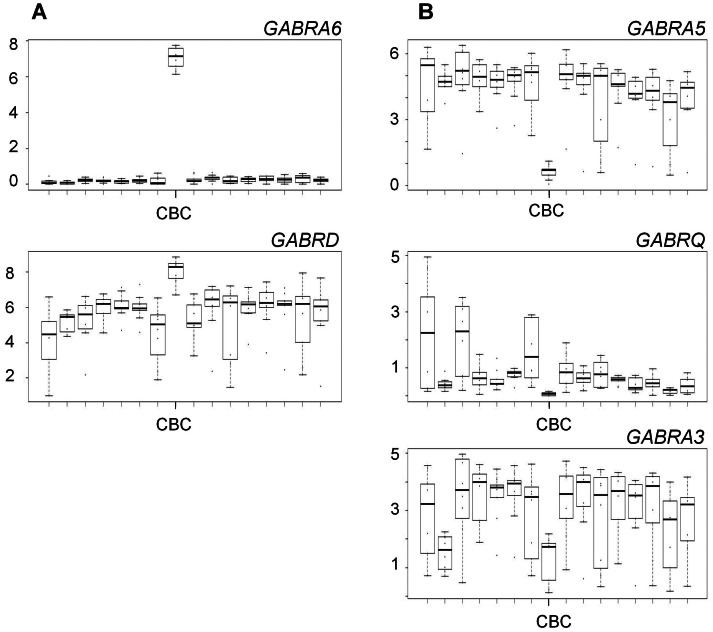
High (Panel A) and low (Panel B) expression cerebellar factors. Panel A: Expression of *GABRA6* is exceptionally high in the cerebellar cortex (CBC) and very low in all other brain regions. *GABRD* is globally highly expressed with the highest expression of all in the CBC. Panel B: *GABRA5* has moderately high global expression but exceptionally low expression in the CBC. The expression of *GABRQ* varies throughout the brain with lowest expression in the CBC. *GABRA3* is moderately well expressed globally with lowest expression in the CBC and striatum. Y axis: gene expression levels are provided as Log2 transformed RPKM (Reads Per Kilobase of exon model per Million mapped reads) values. X axis: the 16 brain regions left to right are the brain regions 1–16 as listed in Table 1.

Three GABA_A_ receptor subunit genes loaded onto this factor that accounted for 0.10 of the total variance in GABAergic gene expression: chr 15 *GABRA5* (1.0); chr X *GABRA3* (0.6) and *GABRQ* (0.5). All these genes have very low expression in the cerebellar cortex (Figure 4).

#### Factor 5: genes with high cerebellar expression

Two GABA_A_ receptor subunit genes loaded onto this factor and accounted for 0.09 of the total variance in GABAergic gene expression: *GABRA6* (1.0) and *GABRD* (0.7). Both genes are highly expressed in the cerebellar cortex (Figure 4); *GABRA6* is not expressed in any other brain region. These genes encode the extrasynaptic α_6_ and δ subunits.

#### Factor 6: GAD2, implicated in GABA synthesis

Only *GAD2* loaded onto this factor (0.7) that accounted for 0.06 of the total variance in GABAergic gene expression. Interestingly, there was also a sub-threshold loading for *GAD1* (0.4).

### Expression of 21 GABAergic Genes in Hippocampal Samples from Alcoholics, Cocaine Addicts and Controls

Using RNA-Seq, expression levels of 17 of the GABAergic genes listed in Table 4 were previously measured in the hippocampus of alcoholics, cocaine addicts and controls from the University of Miami Brain Bank [15]. A pattern of altered expression in cocaine addicts and alcoholics, largely down-regulation, was previously detected [15]. We have now also extracted the *GABRD*, *PRAF2, GABRQ* and *GABRA3* expression values from this previously described dataset [14]. There were significant differences in gene expression for *GABRD* and *PRAF2*:

*GABRD*: F(21,2) = 4.1, p = 0.032; increased expression in alcoholics relative to cocaine addicts, intermediate expression in controls. *PRAF2*: F(21,2) = 3.6, p = 0.044; decreased expression in alcoholics and cocaine addicts relative to controls.

There were no significant differences in gene expression by diagnosis for *GABRQ* and *GABRA3*: p = 0.63–0.25 respectively.

Here we have utilized these data to search for a relationship with the six GABAergic gene expression factors derived from the BrainSpan whole brain data. Our aim was to determine whether some of the factors might show collective changes in gene expression in response to chronic alcohol and cocaine exposure whereas other factors might be unaffected.

### Factors Unresponsive to Chronic Alcohol/Cocaine Exposure

Factor 1: GABA_A_ subunit genes, including the chr 5 cluster: of the 10 genes included in this factor, only *GABRG2*, *GAD1* and *GPHN* showed expression changes in alcoholics/cocaine addicts. It should be noted that *GAD1* and to some extent *GPHN* load more strongly on factor 2.

Factor 4: genes with low cerebellar expression: none of the three genes with low expression in the cerebellar cortex were affected by chronic exposure to alcohol or cocaine.

### Factors Responsive to Chronic Alcohol/Cocaine Exposure

Factor 2: genes involved in synaptic transport: with the exception of *GABBR2* that loads more strongly on factor 1, the remaining six genes all showed altered expression in alcoholics/cocaine addicts.

Factor 3: chr 4 gene cluster: with the exception of *GABRA4* that loads more strongly on factor 1, the other three chr 4 complex genes had altered expression in alcoholics/cocaine addicts.

Factor 5: high cerebellar expression: of the two genes that have exceptionally high expression in the cerebellar cortex, *GABRD* had altered expression in the hippocampus of alcoholics and cocaine addicts. We had no data on *GABRA6* since it is only expressed in the cerebellar cortex.

Factor 6: GABA synthesis; *GAD2*: *GAD2* showed expression changes in alcoholics/cocaine addicts.

## Discussion

It has been known for approximately 20 years that there are four clusters of genes encoding 14 of the 19 GABA_A_ receptor subunits that are located on chromosomes 4, 5, 15 and X [20]–[24]. These subunits comprising the pentameric GABA_A_ receptors are expressed in different combinations in different cell populations and brain regions and differentially during development. There are many examples of clustered genes, for example the hemoglobin and immunoglobulin genes, the major histocompatibility complex and genes relevant to neurobiology such as *MAOA-MAOB* on chr X, *HTR3A-HTR3B* on chr 11 and genes encoding the class 1 alcohol dehydrogenases on chr 4. Gene clusters can arise due to random duplications that offer the possibility of functional diversification. It is thought that the four GABA_A_ gene clusters originated from duplications of (and within) an ancestral gene cluster containing single genes encoding one α, one β and one γ subunit [13]. It is possible that the clustering of genes could enable coordinate transcription resulting in specific receptor subunit combinations. For example, three of the chr 5 cluster genes encode the ubiquitous GABA_A_ α_1_β_2_γ_2_ receptor and three of the chr 4 cluster genes encode the GABA_A_ α_2_β_1_γ_1_ receptor that is found in the mesolimbic reward pathway. Coordinate expression may vary through development, something that we were not able to explore in this study of adults. Linkage disequilibrium provides a mechanism for facilitating coordinate gene expression by maintenance of gene clusters. Although the GABA_A_ gene duplication event is thought to be ancient, yet there is strong linkage disequilibrium across the chr 4 and chr 5 regions, implying selective pressure [1]. Nevertheless, the mechanisms underlying the GABA_A_ gene clustering are currently unknown.

Up to this point information about relative GABAergic gene expression (predominantly of GABA_A_ receptors) has largely derived from preclinical studies, and usually from only a few brain regions. Furthermore, it is important to understand global patterns of GABAergic gene expression in healthy individuals before considering the effects of alcohol and drugs of abuse on GABA neurotransmission. Therefore in this study we accessed data from the BrainSpan Atlas that has been designed as a public resource for studying transcriptional mechanisms involved in human brain development. We were able to perform a comprehensive correlational analysis of the expression of 21 GABAergic genes across 16 brain regions in healthy adults. In order to identify distinct groupings of genes in the correlation analyses we performed a factor analysis which revealed six factors, each of which contributed to >0.05 of the variance and together accounted for 0.86 of the total variance in gene expression. The factor that accounted for one third of the total variance largely comprised genes encoding GABA_A_ receptor subunits. Notably, the highest loading on this factor was for the chr 5 cluster genes that encode the group of subunits comprising the most abundant (60%) α_1_β_2_γ_2_ GABA_A_ receptor. There were also *trans* correlations of chr X, chr 15 and chr 5 genes that together encode the third most common subunit group: α_3_β_3_γ_2_. Considering only the major loadings on this factor, none of the genes, with the exception of *GABRG2*, showed altered hippocampal expression in alcoholics and cocaine addicts. In line with this finding, it is noteworthy that there have been few if any positive association studies between variants in this group of genes and alcoholism (reviewed in Enoch, 2008 [1]).

Three of the four chr 4 cluster genes had major loadings on factor 3. These genes encode the GABA_A_ receptor subunit group α_2_β_1_γ_1_ that is found almost exclusively in the reward pathway. Many human studies have implicated the chr 4 gene cluster in alcoholism and drug addiction (reviewed in Enoch, 2008 [1]). It has been shown that the mRNAs from the chr 4 cluster genes predominate in rat embryo but these genes are generally down regulated in the adult rat except in the hippocampus, the majority of DA neurons in the substantia nigra and the VTA where they are highly expressed [25]–[27]. Indeed, our results showed generally reduced expression of the chr 4 cluster genes relative to the chr 5 cluster genes (Figures S3, S4). In contrast to the factor 1 GABA_A_ receptor subunit genes, all three of the factor 3 chr 4 cluster genes showed expression changes after chronic exposure to alcohol/cocaine [15].

Factor 2 can be regarded as a synaptic transport factor that is strongly influenced by chronic alcohol and cocaine exposure. The five genes that had major loadings on this factor are implicated in GABA synthesis and GABA transport across the synaptic cleft. *GAD1* encodes GAD67, the rate-limiting enzyme for the production of GABA [28]. *SLC6A1* encodes the neuronal GABA transporter GAT-1. *GABBR1* encodes the GABAB1 receptor subunit. Presynaptic GABAB receptors suppress GABA release by inhibiting the activity of voltage-gated calcium channels. *PRAF2* encodes the PRAF2 protein that has been implicated in the control and feedback regulation of synaptic transmission [19]. The fifth gene loading onto this factor, *GPHN*, encodes gephyrin, a scaffolding protein that anchors GABA_A_ receptors to the postsynaptic skeleton. In our earlier study [15] we found that *GAD1, GPHN*, *SLC6A1,* and *GABBR1* were down-regulated in alcoholics and cocaine addicts and we have now shown the same result for *PRAF2* in a re-analysis of the data.

Factor 6 is also implicated in GABA synthesis and is also influenced by chronic alcohol and cocaine exposure. Only *GAD2* loaded onto this factor although *GAD1* had a sub-threshold loading. In mammals, glutamic acid decarboxylase (GAD), the enzyme responsible for metabolizing glutamate to GABA, exists in two isoforms: GAD67 and GAD65 respectively encoded by *GAD1* and *GAD2.* Both isoforms are present in most classes of GABA neurons but GAD67 is present in many cell bodies whereas GAD65 is prominent in axon terminals [29]. It has been shown that GAD67 is the rate-limiting enzyme for the production of intracellular GABA for phasic inhibition [28] and that GAD65 (encoded by *GAD2*) plays a significant role in the synthesis of GABA for tonic inhibition [30].

There were distinct factors for low and high expression of GABA_A_ receptor subunit genes in the cerebellar cortex. Factor 4 included *GABRA5*, *GABRA3* and *GABRQ*, all of which had low expression. The strongest loading was for *GABRA5* (1.0) and as can be seen from Figure 4, *GABRA5* is expressed at moderately high levels in all brain regions except for the cerebellum. Only a small proportion of receptors include α_5_ subunits and these are largely expressed extrasynaptically. Factor 5 included *GABRA6* and *GABRD*, both of which have substantially high expression in the cerebellum. Animal studies have shown that *GABRA6* is only expressed in cerebellum and our study confirms that this is also the case in humans. It has been shown that the α_6_ and the δ subunits are included in extrasynaptic receptors that respond to tonic GABA currents. The cerebellar granule cells appear to have large tonic GABA_A_ extrasynaptic receptor-mediated conductance [31]. Our earlier study showed that the low expression genes loading onto factor 4 appeared to be unaffected by exposure to chronic alcohol and cocaine. In contrast, we have shown that the factor 5 *GABRD* is sensitive to the effects of alcohol/drug exposure. Although we could not show effects on *GABRA6* since it is only expressed in cerebellum, it is perhaps likely to follow the same direction as *GABRD* since both extrasynaptic receptors are potentiated by low doses of ethanol.

One limitation to this study is the fact that we extrapolated the results of the BrainSpan factor analysis derived from the whole brain to the hippocampus (samples from alcoholics/cocaine addicts/controls). This is not ideal however we did not have sufficient data points to perform a factor analysis in hippocampus alone. Nevertheless, it can be seen from figures S3, S4, S5, S6, S7, S8 that for each gene, expression in the hippocampus is similar to expression in nearly all other brain regions. It is only the cerebellum (CBC), and to some extent the mediodorsal nucleus of thalamus (MD) and the striatum (STR) in which some genes show very different expression patterns relative to other brain regions. Whereas it would be questionable to extrapolate the results from the whole brain to the cerebellum, we feel that the evidence provided in figures S3, S4, S5, S6, S7, S8 justify our extrapolation of the factor analysis from the whole brain to the hippocampus.

In conclusion, we have been able to elucidate patterns of GABAergic gene expression across numerous brain regions in healthy adult humans. Although there is a comprehensive understanding of GABAergic gene expression in rodent brain, no such comprehensive studies have previously been performed in humans. Our study has shown that the GABA_A_ gene clusters show both *cis* and *trans* correlations of gene expression. Moreover, the factor analysis revealed that distinct groups of genes, notably those involved in GABA synthesis and synaptic transport and the chr 4 cluster, previously associated with alcohol and drug dependence in humans, are sensitive to the effects of alcohol/cocaine. On the other hand the two factors on which the chr 15, chr X and most of the chr 5 subunit genes loaded were impervious to alcohol/cocaine effects. Although our study was limited to mRNA expression data and cannot necessarily be extrapolated to protein levels, nevertheless our findings might have implications for therapeutic targets to combat stress-related craving and relapse.

## Supporting Information

Figure S1 Relative GABAergic gene expression in human postmortem hippocampal samples from two datasets: BrainSpan and the Miami Brain Bank. The graphs shows mean (S.E.) expression levels for each gene.
(TIF)

Figure S2 Expression of GABAergic genes averaged across all 16 brain regions.
(TIF)

Figure S3 Expression of chromosome 4 GABA_A_ receptor subunit genes in 16 brain regions. The brain regions are identified in Table 1.
(TIF)

Figure S4 Expression of chromosome 5 GABA_A_ receptor subunit genes in 16 brain regions. The brain regions are identified in Table 1.
(TIF)

Figure S5 Expression of chromosome 1 and chromosome 15 GABA_A_ receptor subunit genes in 16 brain regions. The brain regions are identified in Table 1.
(TIF)

Figure S6 Expression of chromosome X GABA_A_ receptor subunit genes and GABA_B_ receptor subunit genes in 16 brain regions. The brain regions are identified in Table 1.
(TIF)

Figure S7 Expression of *PRAF2*, *GPHN*, *GAD1* and *GAD2* in 16 brain regions. The brain regions are identified in Table 1.
(TIF)

Figure S8 Expression of *SLC6A1* in 16 brain regions. The brain regions are identified in Table 1.
(TIF)
